# Dairy, Plant, and Novel Proteins: Scientific and Technological Aspects

**DOI:** 10.3390/foods13071010

**Published:** 2024-03-26

**Authors:** Yaozheng Liu, William R. Aimutis, MaryAnne Drake

**Affiliations:** 1 Department of Food, Bioprocessing, and Nutrition Sciences, North Carolina State University, Raleigh, NC 27695, USA; yliu239@ncsu.edu (Y.L.); bill_aimutis@ncsu.edu (W.R.A.); 2 North Carolina Food Innovation Lab, North Carolina State University, Kannapolis, NC 28081, USA

**Keywords:** dairy proteins, plant proteins, precision fermentation, cell culturing, algal proteins, mycoprotein

## Abstract

Alternative proteins have gained popularity as consumers look for foods that are healthy, nutritious, and sustainable. Plant proteins, precision fermentation-derived proteins, cell-cultured proteins, algal proteins, and mycoproteins are the major types of alternative proteins that have emerged in recent years. This review addresses the major alternative-protein categories and reviews their definitions, current market statuses, production methods, and regulations in different countries, safety assessments, nutrition statuses, functionalities and applications, and, finally, sensory properties and consumer perception. Knowledge relative to traditional dairy proteins is also addressed. Opportunities and challenges associated with these proteins are also discussed. Future research directions are proposed to better understand these technologies and to develop consumer-acceptable final products.

## 1. Introduction

Protein is an essential part of the human diet. In recent years, American consumers have expressed interest in incorporating more protein into their diets [1,2]. Consumers associate high-protein diets with multiple benefits, including high satiety, weight management, weight loss, lipid metabolism, and glycemic regulation [3,4,5]. At the same time, concerns about food sustainability, nutrition, and animal welfare are driving consumers to look for alternative proteins [6]. Alternative proteins are food products that could replace animal proteins [7]. Alternative proteins include plant-based proteins, mycoproteins, algal proteins, cultivated meats, and other protein products [6]. Consumers expect alternative proteins to have lower environmental impacts in terms of lower greenhouse gas (GHG) emissions and less pollution, land use, water use, and biodiversity loss [8]. Accordingly, alternative proteins have gained consumer popularity worldwide [9,10].

Plant proteins have a long history of human consumption. In North America, the European Union (E.U.), and the United Kingdom, the environmental impact of animal agriculture and sustainability started to influence protein decisions from as early as 1971 [11]. The United Nations (U.N.) has linked several of the Sustainable Development Goals (SDGs) closely to food and drink, including zero hunger, clean water, responsible consumption and production, climate, life below water, and life on land, since 2015 [12]. Foley et al. [13] reported that agriculture covers 38% of the earth’s surface and withdraws 70% of the freshwater, while 75% of agricultural land is used for raising animals. If the same land was used to produce plant proteins, it would yield 10 times more than meat and could potentially feed from 10 to 20 times more people [14]. In the U.S., the Environmental Protection Agency (EPA) reported that enteric fermentation from domestic livestock was the largest anthropogenic source of methane (CH_4_) emissions in 2021, accounting for 26.4% of the total CH_4_ emissions (measured in CO_2_ equivalents). Methane emissions were 11.5% of all U.S. GHG emissions in 2021. Accordingly, enteric fermentation contributed to 3.1% of the total U.S. GHG gross emissions (measured in CO_2_ equivalents) in 2021 [15]. Diets shifting away from animal sources have been found to lower the environmental impact [16]. Plant proteins are generally considered by consumers to be more sustainable and ethical and to have less environmental impact than dairy or meat protein [17,18,19].

Food fermentation has a long traditional history, but the revolutionary improvements in targeted fermentation and precision fermentation for specific food proteins have occurred recently. Precision fermentation was first used in the 1980s for human insulin production by the fermentation of recombinant *Escherichia coli* bacteria [20]. After that, the technology was extensively used in the food industry. Since the 1980s, chymosin has been produced by recombinant deoxyribonucleic acid (DNA) technology for use in cheese manufacturing as an alternative to rennet [21]. In 2000, riboflavin or vitamin B2 was produced using genetically engineered *Bacillus subtilis* [22]. Bioengineered *Rhodotorula* (*Rhodosporidium*) *toruloides* has been used to produce lipids and carotenoids [23]. A recent prominent example is the use of an engineered yeast, *Pichia pastoris*, to make soy leghemoglobin to produce plant-based meat (Impossible Foods) [20].

Compared to precision fermentation, cell culturing for protein production is a newer technology. In the food industry, cell culturing is mostly used in cell-cultured meat, although cultured plant cells could be versatile raw materials for future food and pharmaceutical applications [24]. Cell-cultured meat is also called cell-based meat, clean meat, lab-grown meat, and in vitro meat [25]. The first cell-cultured meat was released in 2013 with an exceptionally high cost of EUR 250,000 per Kg. It was a slaughter-free hamburger developed by Professor Mark Post from Mosa Meat [26]. Nowadays, many startups are working on producing cell-cultured meat or seafood using this technology [27,28,29]. However, there are many unknowns, and further research in every aspect of food science and food applications is required.

Algal proteins and mycoproteins (also known as fungal proteins) encompass a variety of proteins and protein ingredients (i.e., bioactive peptides (BAPs)) because of the breadth of alga and fungus species. They have been widely studied by food researchers for years regarding their safety, processing, nutrition, functionalities, and environmental impact [30,31,32,33,34,35]. Previous studies have indicated that algal proteins and mycoproteins have promising benefits for nutrition, health, and sustainability [36,37,38]. Algal proteins have diverse applications in different kinds of food products, while mycoproteins have been used mainly in meat alternatives [39,40]. Up to now, both of these protein categories have had extremely limited applications and commercialization. More research is required to scale up production, improve the product applications, and understand the sensory properties and consumer acceptance of these proteins.

Dairy proteins and plant proteins are popular protein sources, while precision fermentation-derived proteins, cell-cultured proteins, algal proteins, and mycoproteins are novel alternative proteins that are being gradually introduced to the market. Corresponding processing technologies have advanced significantly in recent years. This review will focus on answering the following questions for each protein: What is the definition/composition? How is it manufactured? What is the regulatory status in the U.S. and the E.U.? What is the current market status? What are the safety concerns? What are the functional properties and applications? What are the nutritive values and disease risks? What are the sensory properties and how do consumers perceive these products?

## 2. Materials and Methods

A literature search was conducted in December 2023 to identify eligible articles on the topic of protein ingredients using the JSTOR, PubMed, Web of Science, Agricola, and Google Scholar online databases. The key words used in the search included “dairy/milk protein”, “plant protein”, “precision fermentation protein”, “cell cultured protein”, “algal/algae protein”, and “mycoprotein/fungal protein”. The topics associated with each type of protein included “definition”, “composition”, “processing”, “regulation”, “market”, “safety”, “protein quality”, “nutrition”, “disease risks”, “functional properties”, “applications”, “sensory properties”, and “consumer perception”. Specific inclusion and exclusion criteria were applied to narrow down the search results to relevant articles (Table 1). The PRISMA (Preferred Reporting Items for Systematic Reviews and Meta-Analyses) guidelines were followed [41]. Articles included were peer-reviewed studies published after 2000, published in English, published in scientific journals, and based on primary data.

The literature search identified 6263 dairy/milk-protein-related articles; 8316 plant-protein-related articles; 73 precision fermentation-protein-related articles; 4218 cell-cultured-protein-related articles; 574 algal-protein-related articles; and 4844 mycoprotein/fungal-protein-related articles. These articles were evaluated for their titles and abstracts. A total of 392 articles that met all the predefined eligibility criteria were included in the review. Articles were primarily excluded due to the lack of relevance to protein ingredients or because they focused on subjects unrelated to the topics listed in Table 1.

## 3. Dairy Proteins

Dairy proteins are (primarily) bovine milk proteins and include a variety of valuable dried protein ingredients, including milk proteins, serum proteins (whey proteins), caseins, and other novel value-added ingredients, like lactoferrin, immunoglobulins, etc. [42,43]. Milk proteins consist of a combination of approximately 80% casein and 20% whey protein. Generally, membrane filtration is applied to remove the fat and carbohydrates based on their particle size, and then the protein components are concentrated and dried into higher-protein ingredients. Based on the protein concentrations and different ratios of casein and whey protein, dairy proteins can be classified into ingredients like milk protein concentrate (MPC), micellar casein concentrate (MCC), and milk-derived whey protein (native whey). Whey protein derived from the cheesemaking process is also used to produce whey protein concentrate (WPC) and whey protein isolate (WPI) [44]. A protein concentrate requires a protein content from 25% to 80%, and a protein isolate requires a protein content greater than 90%, according to the U.S. Food and Drug Administration (FDA) [45].

In addition, proteins can be hydrolyzed into peptides and amino acids. Bovine milk is the most studied source for BAPs, and the majority of the current identified BAPs originated from milk proteins [46]. Dairy protein hydrolysates, like casein hydrolysates, caseinophosphopeptides (CPPs), and whey protein hydrolysates, are traditionally produced using enzymatic hydrolysis, but subcritical water hydrolysis (also known as hydrothermolysis) and high-pressure treatments can also be used [47,48,49,50]. Other specific whey proteins have also been isolated and commercialized. Lactoferrin (LF) (2.0–3.3 g/L in human milk and 0.03–0.49 g/L in bovine milk) consists of a simple polypeptide chain [51]. It is known for its health benefits, including lipid metabolism modulation, immune system support, and protection against gastrointestinal tract infections [52,53,54]. Alpha-lactalbumin (α-La), from 20% to 25% of bovine whey protein, also provides immune-modulating effects, antimicrobial activity, antiviral activity, antihypertensive activity, and antioxidative activity [55,56,57,58]. Similarly, immunoglobulins (Igs), the antibodies in milk (about 1.14 g/L in human milk and 0.8 g/L in bovine milk), have multiple immune functions, like promoting the phagocytosis of macrophages against antigens and neutralizing the toxicity of bacterial toxins [59,60].

Dairy proteins are generally recognized as safe (GRAS) based on scientific procedures in accordance with 21 C.F.R 184 [45]. In Europe, dairy products are generally regulated by European Regulation No. 178/2002, which is also known as the General Food Law (GFL) [61]. In addition to the GFL, all non-genetically modified (non-GM) foods that were not consumed in Europe before 1997 are subject to the Novel Food Regulation (NFR), which was adopted in 2015 (EU/2015/2283) [62]. The European Food Safety Authority (EFSA) evaluates scientific evidence, and the European Commission (EC) grants generic novel-food authorizations. Once a novel food has been authorized, anyone can market food products that have the same specifications and use [63]. Dairy protein ingredients usually receive novel-food authorizations, such as whey protein isolates and bovine milk-derived casein hydrolysate [64,65,66]. In general, the global dairy protein market is growing. It was valued at USD 13.8 billion in 2023 and is expected to reach USD 19.6 billion by 2031 at a compound annual growth rate (CAGR) of 5.2% during the period from 2023 to 2031 [67]. The milk BAP segment is predicted to attain a global market size of USD 1.5 billion by 2033, as the global BAP market is growing at a CAGR of 5.3% from 2023 to 2033 [68].

Dairy proteins can be associated with allergy concerns. Milk protein is one of the eight major allergens that account for over 90% of all food allergic reactions [69]. Milk allergy is an adverse reaction to milk proteins, which is medicated by immunoglobulin E (IgE) and can affect the skin (atopic dermatitis or eczema, angioedema, or urticaria), respiratory system (rhinitis, asthma exacerbation, wheezing, pulmonary infiltrates, or acute rhinoconjunctivitis), and gastrointestinal tract (vomiting, recurrent diarrhea, abdominal pain, excessive colic, or esophageal reflux) [70,71]. The major milk allergens are casein (αs1-CN), β-lactoglobulin, and α-lactalbumin, while bovine serum albumin, lactoferrin, and immunoglobulins can also induce milk allergies [72]. The effect of novel-food-processing techniques on milk allergens has been investigated in many studies. Whey proteins (WPCs and WPIs) have shown decreased allergenicity with high-pressure treatment or with the combination of microwave (200 W) and enzymatic hydrolysis [73,74,75]. Meng et al. [76] documented that the structural damage of α-lactalbumin induced by irradiation significantly reduced its potential allergenicity. Hu et al. [77] found that the allergenicity of α-casein was reduced by high-pressure and ultraviolet-C processing. Accordingly, the allergenicity of dairy proteins can be mediated by food-processing techniques. Similarly, concerns regarding lactose intolerance can be solved by filtering out lactose and/or hydrolyzing lactose using enzymes [44].

Dairy proteins are known for their high protein quality and nutrition benefits. The protein quality is a key consideration when selecting protein ingredients. A high-quality protein is defined as a protein that contains all the essential amino acids in the ratios needed by the body while maintaining bioavailability and rapid digestibility [78]. According to international authorities, such as the Food and Agriculture Organization (FAO) of the United Nations, the protein quality is measured using the Protein Digestibility-Corrected Amino Acid Score (PDCAAS) and Digestible Indispensable Amino Acid Score (DIAAS) [79]. The PDCAAS is the percentage of the concentration of the first limiting essential amino acid in the test protein to the concentration of the same amino acid in a reference pattern of essential amino acids (the essential amino acid requirements of the preschool-age child as published in 1985). It is corrected for the true fecal digestibility of the test protein [80]. Proteins with PDCAAS values exceeding 100% are not considered to contribute additional benefits in humans and are truncated to 100% [80]. However, the use of fecal digestibility overestimates the nutritional value of a protein because the amino acid nitrogen entering the colon is lost for protein synthesis in the body and is partially excreted in urine as ammonia [80]. The DIAAS is an improved scoring system and is based on the relative digestible content of the indispensable amino acids and the amino acid requirement pattern [81]. Table 2 compares the protein qualities of different protein sources by listing out the PDCAAS values, which are widely measured in most proteins. Bovine milk proteins have the highest protein quality score with a PDCAAS score of 1 [82] (Table 2). Moreover, dairy proteins are complete proteins, as they contain all the essential amino acids (AAs) [78]. Leucine, one of the nine essential AAs, is a key AA stimulating the initiation of muscle protein synthesis (MPS). Dairy proteins have > 10% leucine contents, while animal proteins generally have 8.5–9% leucine contents, and plant proteins generally have 6–8% leucine contents [82]. Scientific evidence indicates that dairy proteins, especially whey proteins, can stimulate MPS and improve the body composition when combined with resistance exercise or as part of a weight maintenance diet [83,84]. In addition, dairy proteins, including BAPs, have other health benefits, including lowering blood pressure, improving memory, decreasing the viability of cancer cells, and supporting skin health [85,86,87,88,89].

Dairy proteins have good and diverse functionalities and are applied in a great variety of food applications. Milk proteins, namely, whey and casein proteins, are great emulsifiers due to their protein structures. Whey protein is a typical globular protein, while casein has a random-coil structure [106]. Milk proteins have high water-holding capacities, and whey proteins have good solubility, fat-binding, gelling, and whipping properties [107,108]. Protein heat stability is a key consideration for thermal pasteurization. Whey proteins, like whey protein isolates (WPIs), perform better at low pH or under high-acid processing conditions due to their acid stability, which makes them an ideal ingredient in clear ready-to-drink (RTD) applications [109,110]. Casein proteins, unlike whey proteins, have heat stability, allowing for ultra-high-temperature (UHT) processing (142 °C for 3 s) and retort temperatures for protein beverage manufacturing [109]. The heat stability of milk protein beverages in the absence of hydrocolloids or emulsifying salts typically used to enhance the protein heat stability (e.g., clean-label protein beverages) have been demonstrated [111,112]. Accordingly, dairy protein ingredients have wide versatility in food applications, including RTD, low-acid beverages (especially caseins), high-acid beverages (especially whey), ready-to-mix (RTM) beverages, nutrition bars, bakery items, and frozen desserts, as well as soup and sauce applications.

Dairy proteins provide flavor, mouthfeel, viscosity, and structure [42]. Much research has established the flavor profiles and flavor contributions of dairy proteins. Table 3 summarizes the flavor attributes of the major protein types. MPCs with lower protein contents (<70% protein) are characterized by more fluid milk-like flavors, including cooked/milky, sweet aromatic, and a cereal flavor and sweet taste, while higher-protein MPCs and MPIs are characterized by tortilla, brothy, cardboard, and animal flavors as well as higher astringency [42]. Whey proteins (WPCs and WPIs) are associated with sweet aromatic, cardboard, fatty/frying oil, cucumber, potato, cabbage, cardboard, and soapy flavors, a bitter taste, and astringency [110,113,114]. In general, as the protein content increases from skim milk powder to milk protein isolate, the sweet aromatic flavor decreases and the cardboard flavor increases [42,115]. The process of spray drying also increases the cardboard flavor and decreases the sweet aromatic flavor in milk and whey protein [116]. Consumers generally perceive dairy proteins as healthy, affordable, natural, and familiar but less sustainable and ethical than plant proteins [2,19].

## 4. Plant Proteins

**Figure 1:**
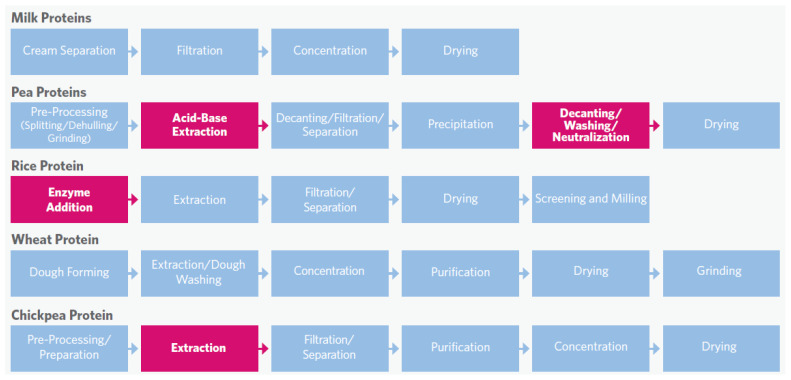
Protein-processing schematics of select protein concentrates made from whole raw materials. Adapted from [155] (with permission).

Plant proteins, or plant-based proteins, are protein fractions extracted from plant sources. These sources can be grouped into cereals (e.g., wheat, corn), edible seeds (e.g., quinoa), pseudocereals (e.g., amaranth, chia), legumes (e.g., pea, soybean), tubers (e.g., potato), oilseeds (e.g., soybean, rapeseed, cottonseed), and algae (e.g., microalgae) [135,136]. In general, the proteins are in the seeds from these plants [137]. Plant proteins are produced by protein extraction, concentration, and purification processes. Plant proteins can be classified into albumins (water-soluble, susceptible to heat coagulation), globulins (soluble in dilute salt solution), prolamins (soluble in 70–80% aqueous ethanol, heat-resistant), and glutelins (soluble in dilute alkali) [138]. In the most commonly utilized plant protein sources, pulses and oilseeds, globulins are the most abundant proteins, accounting for 60–80% of the total protein, followed by albumins, accounting for 10–25% [139]. Because both globulins and albumins have good solubility, they can be extracted with conventional wet-extraction methods [95]. Before extraction, the plant ingredients are usually milled to separate the main components and reduce the particle size by dry milling (pin milling or air classification), expeller pressing (to remove oil), hydrocarbon extraction yielding a meal devoid of oil but retaining protein, or wet milling (grain handling, steeping, separation, and recovery of germs, fibers, proteins, and starches) [140,141,142]. Further extraction is needed to produce protein concentrates or isolates. The conventional extraction processes (wet fractionation) include techniques that use water, salt, solvent, detergent, and alkali, while the protein yield is influenced by the extraction time, solvents, pH, and temperature (Figure 1). To increase the protein recovery and reduce the environmental impact, some unconventional methods (dry and semi-dry fractionation) are emerging, including the use of enzyme, microwave, high-pressure, pulsed-field, homogenization, and sonication methods [143,144,145]. Plant proteins are usually available in two forms: concentrates (50–70% protein) and isolates (>90% protein) [146]. In addition to plant protein powders, plant-based meat alternatives (PBMAs) are produced by the thermoplastic extrusion of plant proteins, and the product types can be categorized as low-moisture (20–35%) and high-moisture (50–70%) [147,148]. Currently, PBMAs are mainly prepared from soy, wheat gluten, and peas [149]. This is because most commercialized concentrates and isolates are extracted from wheat, soybean, rice, peas, and chickpeas, which have strong supply chains and commercial availability [150]. Other sources such as peanuts, rapeseed, oats, rice, lupins, and others are emerging [151,152,153,154].

Plant proteins, including PBMAs, are regulated in a similar manner as other food ingredients labeled as GRAS [156]. Genetically modified (GM) crops like soybeans are also deemed GRAS by both the U.S. FDA [157] and the EFSA with the EC [158]. Certain PBMAs might contain soy leghemoglobin, which is a protein that is produced by GM *Pichia pastoris* and acts as a food additive to provide flavor and color [159]. It has been declared as GRAS in the U.S. [160]. In the E.U., it was evaluated for the potential risk of allergenicity and toxicity in accordance with the Codex Alimentarius Commission 2003/2009 guidelines for genetically modified foods and novel-food ingredients, and it is currently regarded as GRAS in the E.U. as well [161,162]. Currently, plant proteins are consumed as alternative sources of protein in underdeveloped nations and represent a core component of the routine diets in developed countries [163]. The worldwide market value of plant proteins was USD 18.5 billion in 2022 and is expected to reach USD 40.6 billion in 2028 at a CAGR of 14.1% [164].

To meet the growing demand, much research has been conducted in the last two decades on the exploration of novel plant proteins, both extraction and ingredient applications, including novel applications like developing edible packaging and potential therapeutic solutions for energy malnutrition [136,163,165]. Green leaf proteins, including those from alfalfa, amaranth, cabbage, cassava, duckweed, moringa, olive, radish, and spinach, have also received research interest on their compositions, extraction, nutritional profiles, functionalities, and applications [166]. As within the plant protein category, green leaf proteins are GRAS in the U.S. In the E.U., they are subjected to the NFR and require authorizations from the EC; for example, alfalfa protein concentrate received authorization in 2009 [167].

Plant proteins have been extensively researched for their safety, nutritional values, and health impacts. In addition to general microbiological safety concerns, one major safety concern for plant proteins is allergenicity. Food allergies affect 10% of the global population, with a higher prevalence in infants and children [168]. The plant sources that cause allergies are soybean, peanuts, and wheat, but the incidence of allergies to other legumes is increasing, including lentils, peas, and lupins [169]. A number of studies have been conducted to reduce the allergenicity of plant proteins. According to Ding et al. [169], high-hydrostatic-pressure processing can reduce the allergenicity of allergens through different mechanisms, such as protein denaturation, protein aggregation or cross-linking, and protein conformational changes, and it can also inactivate microorganisms. Cold atmospheric plasma-induced protein modification, enzymatic modification, and fermentation have also been shown to alleviate the allergenicity of plant proteins [170,171,172].

In general, plant proteins contain good amounts of essential amino acids, although there is a general deficiency in the amino acid lysine and sulfur amino acids [173]. Accordingly, only soy proteins are traditionally considered a source of complete protein. With advances in plant protein extraction, pea proteins are also considered complete proteins because they contain all nine essential amino acids [174,175]. Plant proteins have poorer proteolytic digestibility and, thus, lower protein qualities because of the presence of fiber and other antinutrition components, like trypsin inhibitors [176]. Most antinutritive factors are primarily found in the cotyledon and hull fractions of legume seeds, while processing techniques can be applied to decrease the antinutritive-factor levels [177]. Overall, plant proteins have relatively low protein qualities, as reflected in their lower PDCAA scores (Table 2), except soy protein concentrates/isolates, which have a PDCAA of 1 [178]. However, plant proteins contain a higher level of bioactive compounds, like phytonutrients (e.g., carotenoids, flavonoids, isoflavones, etc.), which can play an important role in the prevention of several diet-related diseases, such as cancer [179].

Plant proteins are considered functional ingredients because they provide physical attributes in food applications through processes such as solubilization, emulsification, foaming, gelation, and dough formation [180]. Different sources of plant proteins present various functionalities based on their protein compositions and structures [181]. In general, higher concentrations of plant proteins are needed to achieve a comparable functionality to animal proteins [182]. However, deliberate process-induced modifications can be conducted to improve the protein functionality, including enzymatic modification, extrusion, high-pressure processing, high-power ultrasound treatment, etc. [176,179,183,184,185]. Extrusion, particularly, can align the protein fibers and create a meat-like structure [185]. In addition, the modification of soybean functionalities has been studied extensively using pH, ultrasound, enzyme catalysis, pulsed electric fields, etc. [186,187,188,189]. Based on the functionality, plant proteins have been used in a variety of food applications. Pulse proteins have been applied to bakery products, pasta, meat analogues, dairy alternatives, and beverages, and they are being investigated for novel applications, like children’s formula, breakfast cereals, extruded snack products, and BAPs [190]. PBMAs are currently the most common type of meat alternatives due to their approved safety and feasible costs [191]. In addition to plant protein ingredients, plant products like soluble and insoluble fibers and plant protein-derived antioxidant peptides have potential applications in food systems, such as in encapsulation, food packaging, and sensors [192,193]. Electrospun fibers can be incorporated with halochromic compounds or enzymes and act as simple, low-cost, nondestructive, and safe chemo-sensors for food products [192]. Prietto et al. [194] developed a pH indicator from zein and anthocyanins, which changes from pink to green from the acidic to basic condition.

Despite wide use in food applications, plant proteins have major flavor challenges. The off-flavors present in soy proteins are often described as “green”, “beany”, and “grassy” [128,195], while those in pea proteins are often described as, “beany/yellow pea”, “green pea”, “fecal” “grassy”, “cardboard”, and “sulfur” [129,196]. Table 3 summarizes the published flavor attributes of plant proteins. Compared to dairy proteins, plant proteins generally have more off-flavors originating from the specific plant source (Table 3). Accordingly, the flavor of plant proteins and the ingredient applications made with them have remained critical obstacles for consumer acceptability [197]. Different ingredients, like flavor enhancers and bitter-taste inhibitors, can be added during the manufacturing process to improve the flavor [179]. However, this is not ideal for the “clean-label” trend that consumers desire [198]. Clark and Bogdan [199] documented that consumers had negative perceptions and concerns about the high levels of sodium and high degree of processing of plant-based milk alternatives. Chalupa-Krebzdak et al. [200] also documented that plant-based milk alternatives usually have lower protein contents than bovine milk. Other barriers also include affordability and perceived satiety [198]. Despite these barriers, the main drivers of plant-based foods are health, morality, ethics, environmental impact, and animal welfare [201].

## 5. Precision Fermentation-Derived Proteins

Precision fermentation has been named as an emerging food trend in the Fourth Industrial Revolution of the food industry [202]. Precision fermentation is the process of using genetically engineered microorganisms to produce target molecules via fermentation [21]. Precision fermentation products are not labeled as genetically modified organisms (GMOs) as long as they are nature-equivalent and the final product does not contain any GMOs or nucleic acids from the organism(s) used [21,203,204]. Precision fermentation technology minimizes byproduct formation and is a potential substitute for traditional fermentation. In the food industry, precision fermentation is currently used to produce protein and high-value ingredients that originally come from animals. Typically, the microbes are kept in a fermentation tank under conditions that stimulate their growth and multiplication, with optimized temperature, oxygen, light, pH, and nutrient levels. The microbes may excrete the proteins, or cell disruption may be required to release the proteins, which can then be isolated and purified. Although a new technology, precision fermentation has been used to produce enzymes, bioactive compounds, bovine β-lactoglobulin, hen egg ovalbumin, and heme protein, while only precision fermentation-derived β-lactoglobulin and lactoferrin are currently commercially available [159,205,206,207]. Precision fermentation-derived egg white protein has been granted GRAS status in the U.S. [208]. In addition to proteins, it has been used to produce fatty acids, phenolic compounds, like flavonoids, and other food additives, like colorants and flavorings [209,210,211,212].

The cost for precision fermentation has fallen exponentially since the first molecules were produced, and proteins produced by precision fermentation are expected to be at a competitive price point of USD 10/kg by 2025 [213]. Currently, precision fermentation products are mainly used in the healthcare, research, and industrial chemical sectors, with smaller segments in beverage, agriculture, and consumer products. However, it is estimated that the global market for precision fermentation foods and beverages will be worth USD 5.7 billion by 2026 with a CAGR of 51.3% [214]. The prospects for the growing markets for more sustainable and animal-free alternative food ingredients are driving the interest in precision fermentation. Large food and life science companies like DSM, DuPont, Novozymes, and JBS have invested in precision fermentation for developing the alternative-protein industry. However, the interest in employing precision fermentation commercially for alternative proteins has generally been the domain of new startup companies [215].

In the U.S., the FDA has approved precision fermentation-derived proteins as GRAS, such as β-lactoglobulin from the fermentation of *Trichoderma reesei* and soy leghemoglobin from the fermentation of *Pichia pastoris* [160,216,217]. In Europe, the EFSA evaluates the qualified presumption of safety (QPS), which provides pre-assessments of safety risks and publishes a regularly updated list of recommended biological agents for food (including GMOs used for production purposes) and feed use [218]. Safety measures and risk assessment procedures are continuously updated by established organizations like the Food and Agriculture Organization/World Health Organization (FAO/WHO), FDA, and EFSA. In general, if the final product does not contain any GMOs or genetic residues, it falls under the scope of the NFR. If GMOs or residues are present, the premarket authorization is governed by the Genetically Modified Food and Feed Regulation (GMFR) [219]. CRISPR/Cas9, a popular genome-editing tool that can precisely and safely target specific changes in microorganisms without introducing exogenous genetic elements like GMOs [220], is legal in the U.S., but the E.U. still applies the GMO Directive to CRISPR genome-edited organisms [212,221]. Recently, the EFSA concluded that the existing guidelines in the GMO legislation are sufficient to prove the safety of CRISPR genome-edited organisms [221]. They still need to develop suitable documentation and guidelines to regulate CRISPR genome-edited organisms not using the established methods of genetic modification [221,222]. However, there are challenges in marketing using GMO and gene-editing technology for foods because of the public’s lack of knowledge and perceptions of the safety, risks, labeling, and regulation [223,224]. The food industry has explored alternative strategies for developing functional microbes through conventional non-GMO methods [225]. For precision fermentation-derived proteins, the fermentation process favors the use of GRAS microorganisms, which enable simpler GRAS regulatory pathways [214].

Because precision fermentation proteins in most cases have the same molecular structures as their nature equivalents, they have the same allergenicity, nutrition, and functionality as well. For example, individuals who are allergic to bovine β-lactoglobulin would also be allergic to precision fermentation-derived β-lactoglobulin. Precision fermentation-derived β-lactoglobulin would have the same amino acid profile and thus the same protein quality (PDCAAS and DIAAS values) and functional properties as bovine β-lactoglobulin. Currently, there is only one precision fermentation-derived protein commercially available, and that is precision fermentation-derived β-lactoglobulin [216]. It has been used as the main protein ingredient in dairy-free ice creams, cream cheeses, protein bars, etc. [226]. Turtle Tree has self-affirmed as GRAS for their precision fermentation-derived lactoferrin, while Remilk and ImaginDairy have had precision fermentation-derived lactoglobulin approved as GRAS in the U.S. [227,228]. However, bovine whey protein is about 52% β-lactoglobulin, 17% α-lactalbumin, 12% glycomacropeptides, 10% immunoglobulins, 5% serum albumin, 1.5% lactoferrin, and 2.5% other proteins [207]. The single precision fermentation derived β-lactoglobulin does not have the same amino acid profile and diverse protein structure, and thus it does not have an identical protein quality (PDCAAS and DIAAS values) as or similar functional properties to bovine milk proteins or whey proteins [205,229]. Brune et al. [229] investigated substituting cysteine with alanine on the protein structure of precision fermentation-derived β-lactoglobulin to improve its functionality. More research can be conducted to improve the functionality of fermentation-derived β-lactoglobulin, but it would be hard to compete with bovine dairy proteins, which have diverse protein components and structures that contribute to superior functionalities. A further concern is the consumer perception and how to appropriately inform consumers about the difference between fermentation-derived β-lactoglobulin versus bovine milk protein or bovine whey protein.

Precision fermentation proteins face a few major challenges: product yields, upscaling, and consumer acceptance. In order to scale up the process from the laboratory to the industrial scale, further innovation to reduce manufacturing costs is still required. Currently, the scale-up-stage production costs are one of the main bottlenecks of this technology and are prohibitive for many startup companies [20,214,230]. From a technical perspective, more research is needed on process development and optimization to select appropriate growth media, as well as on overproducing strains, substrates, feedstocks, incubation temperature, pH, the suitable fermentation process, and downstream processing in order to increase yields and scale up [231]. Cost-effective and sustainable fermentation feedstocks have been explored, such as byproducts and waste from the agricultural and food industries [232,233]. The downstream purification process can also be very complex [234]. When it comes to consumers, multiple extrinsic and intrinsic attributes can affect consumer acceptance. In general, consumers now look for healthy and sustainable choices that taste good, while the perception of clean labels and naturalness is also important for food acceptability [235,236,237,238]. Many consumers make negative inferences about novel technologies, thinking that they are not in line with expectations for natural, healthy, and tasty foods [239]. Banovic and Grunert [224] found that adopting natural and sustainable framing and prompting the similarity to traditional fermentation could positively influence the consumer acceptance of precision fermentation technology. For specific applications of precision fermentation ingredients in food products, more research on consumer acceptance and consumer perceptions are needed. Thomas et al. [240] reported that 51–61% of survey participants were willing to try precision fermentation-made egg products. To the best of our knowledge, no consumer acceptance studies on fermentation-derived proteins have been published yet. In conclusion, new applications of precision fermentation are driven by both scientific progress and consumer concerns about health, nutrition, and sustainability [20].

## 6. Cell-Cultured Proteins

Cell culturing is another technology that is growing rapidly with the advances in biotechnology. It has been used in the cosmetic and material industries to, for example, produce plant stem cells and leather-like materials, respectively [25]. In the food industry, it is primarily used to produce cell-cultured meat, which is also called clean meat, cell-based meat, or cultivated meat [206]. Cell-cultured meat is genetically identical to conventional meat. However, the structural complexity is challenging to develop in cell-cultured meat, making it difficult to mimic the texture of animal meat [25]. Cultured meat is produced by isolating skeletal muscle stem cells from an animal, inducing cells for proliferation and differentiation in a culture medium, and by engineering tissue structures [241]. Accordingly, cell-cultured meat is distinguished from meat analogs, which have a meat-like texture, color, and flavor but do not contain muscle tissue [135]. One thing noteworthy is that stem cells can be extracted from living animals without the need to slaughter them [206].

The rise of cultured-meat technology is mainly influenced by the development of stem cell biology and tissue engineering, which were initially used in medicine [156]. The first peer-reviewed research on cultured fish was funded by the National Aeronautics and Space Administration (NASA) in 2002 [242]. After that, the first cultured beef burger was debuted in 2013 at Maastricht University [243]. With several years of research and development, economic feasibility is still a major challenge for commercialization. Several researchers have conducted techno-economic assessments of animal cell-cultured meat, but the estimates might not be accurate because they are based solely on the cost of the growth media [244,245,246]. According to Garrison et al. [247], the wholesale cost of cell-cultured meat is estimated to be USD 63/kg, which includes the three major costs of production: the cell culture medium, bioreactors, and labor. Because of the high costs, cell-cultured meats may eventually be competitive and achieve profitability as low-volume, high-value specialty products in the niche market [246,247].

In terms of regulation, the FDA and the United States Department of Agriculture (USDA) will jointly regulate cell-cultured meat in the U.S. The FDA will regulate cell isolation, storage, growth, and maturation. The USDA will monitor products through the remainder of the commercialization process after harvest and oversee labeling [248]. The regulatory process is more complicated if the process involves GM cells or any other DNA manipulations. Labeling is also controversial. There has been an effort from the U.S. Cattlemen’s Association to prevent cell-based products from being labeled as “meat”, although the North American Meat Institute has stated that cell-based products likely fall into the definitions of either “meat” or “meat byproduct” [156,249]. For Europe, cell-cultured meat could be applicable to the E.U. NFR pathway, while GM food will be regulated differently in different countries. The EFSA has approved GM food production after thorough safety assessments, but many European countries, like France and Germany, have banned GM foods [250].

Potential chemical and biological hazards along the steps of the manufacturing process have been identified by 87 cell-cultured meat and seafood industry representatives and researchers [251]. These hazard considerations cover microbiological contamination, the health status of the source animal, the cell culture medium, antibiotics, cryoprotectants, physicochemical transformations, GM and novel expression products, adherent surfaces and dissociation reagents, and other chemical substances [251]. Methods to control these hazards include Good Manufacturing Practice (GMP), Good Cell Culture Practice (GCCP), the code of hygienic practice, hazard and risk management systems, input material and equipment selection, and contaminant control [251]. For the final product, multiple analyses could be conducted to ensure safety. Theoretically, cell-cultured products are biochemically, genetically, and compositionally similar to existing foods and should be as safe as their conventional counterparts [251]. Accordingly, cell-cultured products should have the same allergenicity and nutrition as their conventional counterparts, even though the functionality might not be the same. Cell-cultured products might not have the same water-binding and -holding properties as intact muscle. The final cell-cultured meat or seafood product usually requires food additives to improve its flavor and texture (e.g., flavorings, stabilizers, etc.), which might induce allergy and nutrition concerns.

Consumer acceptance of cell-cultured meat has been studied widely even though commercial products are not widely available. In general, consumers have various attitudes towards cell-cultured meat. Wilks and Phillips [252] found that U.S. vegetarians and vegans were more likely to agree with the potential benefits of cell-cultured meat but were less willing to try it compared to omnivores. Vegetarians and vegans usually have greater concerns about food sustainability but may oppose GM foods and have remaining concerns regarding animal welfare and cultured meat [202,253]. Zhang et al. [254] discovered that Chinese consumers had limited knowledge of cultured meat, but more than 70% of urban consumers were willing to taste or purchase it. In practice, many factors affect consumer purchase decisions, including sensory properties, psychological factors, marketing factors (price, brand), labels (origin, local), etc. [255]. The most common concerns with cell-cultured meats are the unnaturalness, safety, healthiness, taste, texture, and price [256]. Siegrist and Sütterlin [257] reported that a lack of naturalness reduced the acceptance of cultured meat for European consumers, even with their awareness of the potential environmental and animal welfare benefits. Tucker [258] stated that many consumers believed cell-cultured meat had poor flavor, texture, and color compared to conventional meat. Similarly, Bekker et al. [259] and O’Keefe et al. [260] documented that consumers had similar expectations for cell-cultured meat as for conventional meat. Consumers also believed cell-cultured meat should be less expensive than conventional meat [260]. Rolland et al. [261] reported that 58% of consumers who tasted conventional burger but thought it was cell-cultured meat would pay a 37% premium for the cell-cultured meat over the regular meat in a study emphasizing the role of positive information in enhancing the consumer acceptance and willingness to try cultured meat. In addition, nomenclature affects consumer acceptance. For example, “animal-free” and “clean” incited more positive attitudes than “lab-grown” in consumers [262]. Accordingly, the major challenges of cultured meat are commercialization scale-up, regulation and labeling, and consumer acceptance. Lee et al. [197] suggest that meat alternatives will be part of future protein sources. They will remain a complement to traditional meat but will hardly replace it because of the difficulties in the technical development and the challenges in consumer acceptance.

## 7. Algal Proteins

Algae are defined as a diverse group of species that are oxygen-producing, photosynthetic, unicellular or multicellular organisms, excluding embryophyte terrestrial plants and lichens [263]. Algae include macroalgae (seaweed) and microalgae. Macroalgae can be divided into three main groups based on their color: *Phaeophyta* (brown algae), *Chlorophyta* (green algae), and *Rhodophyta* (red algae) [264]. Microalgae are unicellular, microscopic organisms and have been estimated to include ~350,000 species, with only ~10–100 of these being well researched [265]. The most abundant microalgal divisions are *Bacillariophyta* (diatoms), *Chlorophyta* (green algae), *Chrysophyta* (golden algae), and *Cyanophyta* (blue-green algae) [266]. Well-known protein-rich microalgae species include *Arthrospira*, *Chlorella*, *Aphanizomenon*, and *Nostoc* [267,268]. Algae are rich in protein, vitamins, minerals, dietary fiber, and bioactive compounds [36]. Algae are considered a viable and sustainable source of protein. Macroalgae have a protein content ranging from 9 to 47%, and microalgae typically have a protein content as high as 70% [30]. Algal proteins have been noted for their applications in animal feed, food, and aquaculture for many years. Fowden [269] studied the compositions of protein fractions from different algal classes and concluded that the distributions of amino acids were similar among different algal species. Taub and Dollar [270] investigated using light to increase the protein yield in the alga *Chlorella*. Mayfield et al. [271] studied the efficient expression and assembly of a fully active antibody in *Chlamydomonas reinhardtii*. The large-scale cultivation and production of algae and algal proteins are usually achieved with two approaches: enclosed bioreactors with close control of the environmental parameters and operation conditions, or low-cost open units like ponds that are unmixed or mixed by paddle wheels, pumps, or air-lift systems [272,273]. In addition, an alternative to photobioreactors and a potential means for substantially reducing growth costs is to use conventional fermenters to grow heterotrophic algae by culturing with a carbon source, like glucose [274]. In addition to incorporating the whole algal biomass into food, algal protein isolates can be extracted using a variety of methods, such as solvent extraction, bead milling, high-pressure homogenization, subcritical water extraction, and pulsed-electric-field-assisted extraction followed by purification using ultrafiltration or ionic-exchange chromatography [32,275,276,277,278]. After that, BAPs, currently the most commercially attractive microalgal products, can be produced using chemical hydrolysis, enzymatic hydrolysis, microbial fermentation, or other techniques based on their molecular sizes or charges [279,280].

The consumption history of an alga affects its regulatory status. In addition to *Arthrospira* and *Chlorella*, which have been designated as GRAS, most microalgae and novel microalgal products, like microalgal proteins, lipids, and BAPs, are subjected to the NFR in the E.U., and a number of microalgal products have been approved by the E.U. [281,282]. In the U.S., *Arthrospira*, *Chlorella*, *Crypteconidium*, *Dunaliella*, and *Haematococcus* have been notified as GRAS, while other microalgae relevant for food or feed applications are subjected to premarket review and approval by the FDA [268]. Algae have been gaining popularity as “superfoods” across food and beverage categories as main ingredients, flavoring agents, or natural colorants in premium-product launches [283]. In 2023, the global market of algal products was estimated at USD 5.3 billion, and it is projected to grow at a CAGR of 6.4% to reach USD 7.3 billion by 2028 [284]. Moreover, the global algal protein market size was worth USD 3.2 billion in 2021, expanding at a CAGR of 8.4% from 2022 to 2030 [285].

Algae are associated with a few safety risks. Because marine algae are exposed to radioactive pollution and plastic pollution in the ocean, they can have potential radioactive contamination. In 2014, Japan’s Fukushima nuclear power plant accident resulted in radiation levels that exceeded the safety limits in most marine algae, especially edible seaweeds [286]. Radioactive substances like cesium can be measured and monitored to prevent radioactive contamination [287]. In addition, algae might also be associated with excess intakes of iodine, heavy metals, and pesticide and veterinary drug residues [31,288,289,290]. Iodine is an essential trace element for the human body, but an excess intake of iodine can result in an increased prevalence of hypothyroidism and increased risk of thyroid cancer [291,292]. In general, the recommended intake is 150 micrograms per day for adults [293]. At present, research on the detection of these chemical factors affecting the food safety of algae is updated constantly [294]. Moreover, certain species of algae may contain allergens and toxins. Allergenicity has been reported for the airborne cyanobacteria *Phormidium fragile* and *Nostoc muscorum*, and for the green algal genus *Chlorella*, although a high-lipid product composed of dried, milled *Chlorella protothecoides*, Whole Algalin Flour, showed little potential for food allergy [295,296]. With regard to toxins, toxic microcystines have been detected in *Aphanizomenon flosaqua* [297]. Toxic prostaglandins (PGE2) are found in *Gracilaria vermiculophylla* [298]. No toxins have been found in *Spirulina* or *Chlorella* [299]. Another safety aspect is the presence of pathogens. Microbial contamination from *Salmonella*, *Bacillus*, pathogenic *Escherichia coli*, *Listeria*, *Staphylococcus aureus*, or Vibrio can occur during the cultivation, harvest, and handling of macroalgae [300,301]. However, this issue is more susceptible to raw-seaweed products instead of processed algal-protein ingredients.

Algal proteins contain all the essential amino acids and are therefore complete proteins [302,303]. Information on the PDCAAS and DIAAS values of algal proteins is extremely limited. The published PDCAAS values of algal species are listed in Table 2. No information about the DIAAS values of microalgal protein products for human foods is currently available [304]. Based on the published values, the PDCAAS values of algal proteins are generally less than 1. They are lower than dairy proteins and are comparable to plant proteins (Table 2). The PDCAAS values of the microalgae *Chlorella vulgaris* and *Chlorella sorokiniana* are higher than those of pulses such as lentils, beans, peas, and chickpeas [305]. The protein digestibility of algal proteins is inhibited by the high fiber and lectin contents and the presence of polysaccharides and other compounds, like polyphenols [306,307]. The digestibility of algal proteins can be enhanced by cell wall disruption, fermentation, and enzymatic processes [97,308,309]. Algal proteins are a good source of BAPs, which present antioxidant properties, antihypertensive properties, anticoagulation properties, cancer suppression, immune stimulation, and so on [310,311,312,313]. In addition, algal polysaccharides, some considered as dietary fiber, have health benefits such as antibacterial and anti-inflammatory effects and a low caloric index [314,315,316].

Algal proteins present diverse functional properties. Microalgal protein extracts have low solubility for acidic pH close to their isoelectric points and high solubility in neutral or basic environments (pH > 6.5) [317]. However, Grossmann et al. [318] used high-pressure homogenization to disrupt *Chlorella protothecoides* cells and obtain soluble proteins with a high solubility of >90% between pH 2 and 6. The soluble *Nannochloropsis oceanica* protein fraction also has high solubility that can be useful in acidic, low-viscosity food [319]. Algal proteins are good emulsifiers due to their polysaccharide and phospholipid contents [320]. They can form stable emulsifying complexes that can be used in food applications, such as protein–pigment complexes [321]. Microalgal proteins present comparable emulsification properties to those of soy, egg white, and dairy proteins. The emulsion stability (ES) of *Tetraselmis* sp. protein isolates was better than those of whey and egg protein isolates [322]. Soluble proteins of *Chlorella vulgaris* had a better emulsifying capacity (EC) than soy protein isolates and caseinates [323]. In addition, algal proteins present excellent gelling properties [33,324]. Algal proteins from some species present high foaming capacities (FCs) and foaming stabilities (FSs). Soluble protein extracts of *Tetraselmis* sp. formed highly stable foams that were comparable to soy proteins, whey proteins, and egg white albumin over a pH range of 5.0–7.0 [322]. Soluble proteins from *Chlorella pyrenoidosa* had high FCs (>95%) with FSs over 180 min [325]. Accordingly, algal protein can have diverse applications in a variety of food products, such as snacks, drinks, pasta, cookies, baking items, sauces, meat substitutes, and ice creams [326,327,328]. However, algal proteins currently have low commercialization and marketability. The major challenges are the lack of scalable and cost-effective algal cultivation and knowledge gaps regarding the harvesting and downstream processing [267,329]. The other challenge is the lack of research on food applications [268].

The sensory properties of algal protein vary depending on specific species and applications. Generally, algal proteins have strong flavors and colors, particularly a green color, fishy aroma, and seaweed-like taste [330]. Algae usually have a strong umami taste, and some may have a bitter taste (e.g., *Phaeodactylum tricornutum*) [331,332]. Four classes of typical odor-active volatile or taste-active non-volatile chemicals in algae are known: fatty acid-derived volatile compounds (aldehydes, alcohols, and ketones), sulfur compounds (dimethyl disulfide, methanethiol, etc.), nitrogen-containing compounds (trimethylamine, etc.), and umami-tasting compounds (glutamate, aspartate, inosine monophosphate, etc.) [331,332,333]. *Chlorella* sp. have strong vegetable-like flavors with an intense green color [334]. *Tetraselmis suecica* and *Phaeodactylum tricornutum* marine algae have been reported to have an intense fishy flavor [335]. *Spirulina* extrudates were reported to have a black color and intense earthy and algal flavors [336]. Strong fishy flavors are typically not appreciated in applications like cookies, yogurt, pasta, etc. [335,337,338]. However, they can serve as natural flavoring and coloring agents in fish/marine products, sauces, and meat and fish analogs, in addition to providing nutritional and functional benefits [339,340,341,342,343]. In addition, the off-flavors can potentially be mitigated by physical methods (i.e., ultrasound, ultrafiltration, etc.), chemical methods (i.e., antioxidant treatments, Maillard reaction, etc.), or a combination of different methods (i.e., activated carbon adsorption–ultrafiltration, encapsulation–adsorption, etc.) [344]. Michel et al. [345] reported that consumers had positive opinions on algal meat analogs because of the nutritional and environmental advantages, but they had low taste expectations. Van der Stricht et al. [346] found that E.U. consumers were unfamiliar with food products with microalgal proteins but were willing to try them. They identified four consumer cluster profiles: “Enthusiast”, “Cautiously Curious”, “Currently Waiting”, and “Uninterested” [346]. Similarly, Mellor et al. [347] stated that British consumers had limited knowledge of algae as a food source but were willing to try them, and the anticipated acceptance of algae was influenced by the perceived novelty, edibility, healthiness, sustainability, and affordability. Weickert et al. [348] investigated the consumer acceptance of microalgal cultivation technology. Neophobia and information frames did not affect German consumer acceptance of the microalgal technology [348]. Lafarga et al. [349] reported that Spanish consumers considered microalgae sustainable, nutritious, and safe, while the main reasons for low consumption were lack of knowledge and lack of habit. In general, increasing the consumer awareness and knowledge of algae and algal proteins could increase the market shares of algal products [346,347,349]. However, flavor (aromatics and basic tastes) remains a critical barrier.

## 8. Mycoproteins

Mycoproteins, or fungal proteins, are derived from filamentous fungi. *Fusarium venenatum* is the main strain used to cultivate and harvest mycoproteins [34]. In the 1960s, concern regarding future global protein shortages due to the projected population expansion prompted food researchers to try to create a palatable, affordable source of microbial protein [350]. After analyzing more than 3000 fungal isolates from around the world, *F. venenatum* A3/5 (ATCC PTA-2684) was eventually selected to be the best strain to produce mycoprotein because it does not produce toxic microbial metabolites [351]. Accordingly, mycoprotein nowadays is the generic name given to the ribonucleic acid (RNA)-reduced biomass comprising the hyphae (cells) of mostly *F. venenatum* A3/5 (ATCC PTA-2684) produced in the continuous-fermentation process [350]. Currently, mycoproteins are produced by fermenting agro-industrial waste, such as seaweed waste, soy waste, pineapple peel waste, pea process byproducts, and date waste [38,352,353,354]. The microbial fermentation can be carried out by submerged fermentation, solid-state fermentation, or the surface culture method [37,355,356]. Submerged fermentation has been proven to produce a higher yield and more nutritional benefits [35,357,358]. In addition to *F. venenatum*, several other fungi have also been employed for mycoprotein production using different fermentation methods, such as *Pleurotus albidus*, *Neurospora intermedia*, *Rhizopus oryzae*, and *Aspergillus oryzae* [38,359]. A life cycle assessment (LCA) conducted by Upcraft et al. [360] indicated that mycoprotein production produces less gas greenhouse emissions compared to plant and animal proteins. Compared to beef, pork, and chicken meat, mycoprotein production involves a smaller carbon footprint, fewer nitrogen emissions, and less water use and land use [361,362,363].

Mycoprotein produced from *F. venenatum* A3/5 was approved for trade as food protein by the Ministry of Agriculture, Fisheries and Food (MAFF) in the United Kingdom in 1984 and is available for purchase in all E.U. member states [364]. During this time, the British company Marlow Foods, a joint venture of Rank Hovis McDougall (RHM) and Imperial Chemical Industries (ICI), was solely responsible for the commercialization of mycoprotein, and they made extensive use of mycoprotein in vegan and vegetarian food applications under the brand Quorn™ [365]. Mycoprotein was designated as GRAS by the FDA in 2002 [157]. After that, it was permitted for sale in the U.S., Norway, Australia, Switzerland, and, more recently, Canada, Thailand, Japan, and Malaysia. Nowadays, Quorn, founded by Marlow Foods and now owned by Monde Nissin Corporation, is still the largest mycoprotein manufacturer in the world, operating in approximately 20 countries [40,366]. The Quorn fermenters use submerged fermentation and are the largest continuous-flow culture systems in use by the biotechnology industry worldwide [367]. The other major mycoprotein manufacturers include 3F Bio, General Mills, MycoTechnology, Temasek Holdings, and Tyson Ventures. The global market of mycoprotein was estimated to be USD 642 million in 2022 and is projected to be USD 1.1 billion in 2030 with a CAGR of 6.4% [368].

Although mycoproteins are generally safe to consume, they can potentially cause allergic and gastrointestinal symptoms [147,369,370]. Jacobson and DePorter [371] analyzed 1752 self-reported adverse reactions associated with mycoprotein (Quorn-brand)-containing foods, which included urticaria, anaphylaxis, nausea, emesis, and diarrhea. Because mycoproteins share multiple common allergenic determinants with *Aspergillus fumigates* and *Cladosporium herbarum*, and some with *Alternaria alternate*, mold-allergic patients might also have reactions to mycoprotein [147,372]. These incidences appear to be extremely low. Finnigan et al. (2019) [373] analyzed that the frequency of possible allergic reactions to mycoprotein was one per 24.3 million servings in the U.K. between 2003 and 2017. Another safety concern might be mycotoxins, but the strains used in mycoprotein production usually do not produce mycotoxins, and mycotoxins are assessed in safety tests [374,375]. In general, mycoproteins have minimal properties of intolerance [376,377].

Mycoproteins have multiple nutrition and health benefits. Mycoproteins are a good source of complete protein and have high protein quality (PDCAAS = 0.996), high fiber contents, and reduced saturated fatty acid contents [37,378]. Accordingly, mycoproteins have a similar protein quality to that of animal and dairy proteins. In addition, mycoproteins have been reported to improve appetite regulation by the regulation of metabolic hormones, satiety, and blood sugar and cholesterol levels, as well as to reduce cardiovascular diseases, provide antihyperlipidemic, antioxidant, and antimicrobial activities, and stimulate muscle protein synthesis [379,380,381,382,383,384,385,386].

Mycoproteins have great gelling, emulsifying, and foaming properties [387,388,389]. A range of fungal cell membrane lipids, including nucleotides and nucleosides, sterols and sterol esters, phospholipids and lysophospholipids, monoglycerides, diglycerides, and their derivatives, are widely used in the food industry due to their gelling, emulsifying, foaming, and thickening properties [390]. The heating steps during fermentation can reduce the RNA content, resulting in higher levels of fungal filament entanglement, which can contribute to enhanced rheological properties [389]. Mycoproteins are primarily used in meat alternatives [374,391,392]. Mycoproteins have been used to prepare minced meat, chicken pieces, burgers, sausages, nuggets, fillets, ready-to-eat meals, cakes, pies, etc. [35,350]. Moreover, mycoproteins are also widely used in frozen-food products [40].

Sensory and consumer studies of mycoprotein are extremely limited. In general, the flavor of mycoprotein can be affected by the choice of microorganism, while less flavorful strains can be flavored with supplemental materials and are valued more for their textural contributions to the food product [393]. Shahbazpour et al. [394] compared the oxidative stability, texture, and color of cooked beef sausages and mycoprotein sausages and concluded that the mycoprotein substitution improved the nutritional and health effects of the sausages but resulted in reduced textural characteristics, such as the hardness, cohesiveness, gumminess, and springiness indexes. Elzerman et al. [395] compared Quorn pieces and minces. Quorn pieces were more acceptable than Quorn minces in individual evaluations when included in meal salads and rice dishes, and both meat substitutes were widely accepted in soups and spaghetti dishes [395]. Elzerman et al. [395] pointed out that more emphasis is needed on the consumer evaluation of meal combinations instead of on the sensory properties of the individual product. Hashempour-Baltork et al. [35] documented that their formulated mycoprotein nugget had similar texture attributes (hardness, springiness, cohesiveness, and chewiness) to those of chicken nuggets (*p* > 0.05). In terms of consumer perception, Dean et al. [396] reported that the largest driver of the consumer willingness to consume mycoprotein was healthiness, followed by the nutritional benefits, consumption safety, and sustainability. Chezan et al. [397] found that sensory attributes were the most important factor in the acceptance of meat substitutes, while consumers also valued clean-label products. The overall acceptance of mycoprotein was low, which might be due to the low familiarity with it as well as the perceived low appeal and lack of tastiness of the available fungal-protein products [397]. Overall, more research is required to better understand the consumer acceptance and perception of mycoprotein products.

## 9. Discussion

Dairy proteins generally have higher protein qualities than plant proteins based on the PDCAAS and DIAAS values. Dairy proteins are complete proteins, while most plant proteins are not complete proteins. Dairy proteins also have diverse functionalities, mild sensory properties in comparison to plant proteins, and, therefore, more diverse applications. In contrast, plant proteins are inferior in these attributes and require further modifications and processing in applications. Off-flavors like beany and green pea remain in the final products due to the nature of the plant protein composition. Lactic acid bacteria (LAB) fermentation can improve the flavor and biological availability of nutrients in cereal-, pseudocereal-, and legume-based beverages [398,399], but flavor remains a barrier in many plant protein applications. Lower functional properties, such as lower solubility and heat stability, may necessitate the use of additional ingredients in the final application, such as shelf-stable RTD beverages made with plant proteins. The current consumer desire for clean labels may be an advantage that the dairy industry should carefully consider. Although consumers generally perceive dairy proteins as less sustainable than plant proteins, more research on life cycle assessment is still required. Dairy proteins have many viable co-products, like lactose, minerals, etc., that have value-added uses in food applications [155]. Moreover, dairy proteins can be produced sustainably through cow care and management as well as improved dairy productivity. From 1950 to 2017, advancements in farming and management practices enabled dairy farmers to produce 79% more milk while milking 59% fewer cows (from 22 million cows to 9 million cows), accounting for a 66% smaller carbon footprint [400]. In addition, 80% of cow feeds are not digestible by humans, meaning that cows are converting nonviable food sources to viable food sources. In the 20% of cow feeds that are digestible by humans, only 2% are edible by humans [401]. Consumers generally lack knowledge on basic food nutrients, animal nutrition, and food processing and need to be better informed, which might mean marketing opportunities for the dairy industry.

Among all the alternative proteins reviewed in this paper, plant proteins in predominantly meat and dairy alternatives have the longest consumption history, resulting in more market availability, better regulation, and higher consumer acceptance than other emerging types of proteins. However, emerging proteins address or complement some plant protein characteristics. For example, algal proteins present a wide range of nutrients as well as bioactive compounds but face similar challenges as plant proteins in sensory acceptance due to their color and strong “sea” flavors. However, algae have higher growth and production rates and higher photosynthetic efficiency, consume less water, do not compete for arable land, facilitate extraction processes, and can be sustainably cultivated [402]. Similarly, mycoprotein has a reduced environmental impact compared to those of dairy and plant proteins and has high nutritional and health benefits. However, mycoprotein is primarily used in alternative meat products and requires more research on its product development, sensory properties, and consumer perception. Both precision fermentation and cell culturing are essential parts of cellular agriculture and require an extensive amount of knowledge and research in biotechnology and processing technologies. The difference between these two protein sources is that precision fermentation proteins are specific proteins produced by GM microbes grown under controlled fermentation conditions, while cell-cultured proteins refer mostly to cell-cultured meat or seafood starting from animal tissue, although there are startups currently developing cell-cultured milk components (Biomilq), cell-cultured collagen (Jellatech), and cell-cultured gelatin (Perlita). Cell culturing involves growing stem cells in bioreactors to produce animal muscle tissues, but, to date, only non-structured cell masses have been harvested. Commercial production of both protein products is limited at present. Therefore, opportunities exist to use cell masses in conjunction with plant-based or actual animal proteins to commercially offer alternative meats (hybrid products). More regulatory conversations need to occur for the adequate regulation and labeling of these products, and more research is needed to understand their safety, nutrition, disease risks, manufacturing processes, sensory properties, and consumer acceptance. Currently, precision fermentation can only successfully produce specific protein and/or bioactive components, and cell culturing is solely the proliferation of the stem cells or starter tissue. Animal proteins, including dairy proteins, are extremely complex and have various protein components. Therefore, precision fermentation-derived proteins and cell-cultured proteins are inferior to animal proteins and require more processing and food additives in applications to match the nutrition, functionally, and sensory properties of animal proteins. Although algal proteins and mycoproteins are complete proteins, they also have lower protein qualities than dairy proteins. Additionally, more research and process optimization are required to improve their functionality, sensory properties, and food applications.

Among all alternative proteins, one common and major challenge to consumer acceptance is the poor sensory properties. Schouteten et al. [403] reported that taste was the key factor inhibiting the consumption of plant proteins in Germany, the Netherlands, and France. In addition, plant proteins have limited use in food applications due to their restricted functional properties, like their tendency to aggregate, low solubility, and low heat stability. Similarly, algal proteins generally have strong flavors and colors, which are undesirable to consumers [330]. Mycoproteins can have an earthy flavor depending on the choice of microorganism [393]. For precision fermentation-derived proteins, many patents have been filed to make ingredients like recombinant enzymes, animal-free egg replacers, recombinant milk proteins, recombinant collagen, and recombinant heme proteins to provide color and flavor to PBMAs [159,404,405,406]. However, there is little research addressing the sensory properties of these ingredients and/or their applications. Although precision fermentation-derived proteins have the same primary amino acid sequence as their natural equivalents, the specific components used and their contacts between food molecules influence the functional, nutritional, and sensory properties of food [407,408]. Furthermore, precision fermentation proteins lack post-translational modifications (e.g., phosphorylation or glycosylation) that could impact functionality in food systems [214]. In terms of cell-cultured proteins, many consumers anticipate cell-cultured meat or seafood to have an inferior taste, texture, or appearance compared to their conventional counterparts [258,259,409,410]. Additional food additives can be added into cell-cultured meat to improve the flavors and other sensory properties [411,412]. Cultured muscle tissues generally have a pale color due to the absence of myoglobin [413]. Myoglobin added to culture media has been found to increase the proliferation and improve the coloration of cell-cultured meat [413,414]. Similarly, as fat is crucial for aroma, juiciness, and tenderness, multiple techniques can be used to potentially enhance the fat-related flavor sensory properties, such as the co-culturing of different cell types, scaffolding, medium supplementation, genetic modification, etc. [156,412,415]. Scaffolding is a method used to build a more meat-like texture in cell-cultured meat and seafood. It is a diverse technology adapted from tissue engineering to support cell attachment and proliferation [412,416]. Currently, scaffolding is only capable of producing ground and emulsified cell-cultured meat or seafood products, and more improvement is needed to produce highly developed structures of cell-cultured products [417]. To produce steaks, whole cuts, and other complex tissues, bioprinting, the use of material transfer processes for patterning and assembling biologically relevant materials with a prescribed organization, has also been investigated as a possible structuring technique [418,419]. Notably, precision fermentation can be used to transform or optimize the manufacturing process of cell-cultured meat or seafood because precision fermentation offers serum-free media for cell proliferation, produces compatible biomaterial for scaffolding, and can provide antioxidants and anti-freeze proteins for cell-cultured products [420]. In summary, regardless of the alternative-protein type(s) used, the ultimate goal is the creation of protein products with good flavors, textures, and appearances that are safe, nutritious, and healthy, as well as affordable and convenient.

Alternative proteins are all associated with benefits for sustainability, including less GHG emissions, less resources used, and animal welfare. They are usually advertised to consumers emphasizing these benefits. As a result, consumers perceive plant proteins and related products to be eco-friendlier and more natural than animal proteins [421,422,423,424]. However, data quantifying the environmental and sustainable benefits are very limited, especially for protein concentrates and isolates. Some consumers are skeptical about the environmental benefits as well [425,426]. For example, Switzerland consumers evaluated meat substitute products as less environmentally friendly and less heathy than meat [425]. Some studies have investigated the environmental impact of plant proteins, suggesting that plant-based products generate less GHG emissions and use less water and land than animal products, while the most common measure is GHG emissions or the global warming potential [427,428,429,430]. For cellular agriculture, the potential ecological benefits could be countered by increases in agricultural activity in other regions for the production of feedstock. Therefore, the broader impact on individuals, communities, and the environment should be considered and investigated to understand whether cellular agriculture aligns with the sustainability goals [431,432]. As a result, more research is required to develop relevant assessment methods and policies and assess the environmental impacts of each technology type and protein product.

## 10. Conclusions

This review summarizes scientific and technological aspects of dairy proteins, plant proteins, precision fermentation proteins, cell-cultured proteins, algal proteins, and mycoproteins. Opportunities and challenges for each protein type were comprehensively analyzed, which can provide insights for future research and development. Dairy proteins have good protein qualities, good nutrition, functionality, and sensory properties, and versatile applications. They have complex protein components, resulting in various value-added protein ingredients and co-products, and they can be produced sustainably. However, consumers need to be better educated in science-based information. More research can be conducted to further improve the heat stability and reduce the environmental impacts of dairy proteins. Alternative proteins are growing fast and have gained popularity globally as consumers look for foods that are healthy, nutritious, and sustainable. The consumption of plant proteins has a long history, and they are regulated similarly to other foods. In addition, the safety, nutritional values, health impacts, and functionalities of plant proteins are generally well established. However, plant-based meat alternatives are a relatively new segment and need more research and development to produce desirable products that are comparable to conventional meat products. Both precision fermentation and cell culturing are crucial cellular agricultural technologies. Precision fermentation is mainly used to produce high-value protein products and functional ingredients, while cell culturing is solely used to produce cell-cultured meat or seafood. Precision fermentation and cell cultivation have been active for several decades and one decade, respectively. Given that both technologies are relatively new to the food industry and require more research on the manufacturing process and upscaling, commercial products are very limited. Similarly, the commercialization of algal proteins and mycoproteins is extremely limited. More research on upscaling, reducing costs, food applications, the sensory properties, and consumer perception is needed. Many opportunities and challenges are presented to eventually produce successful products that are tasty, healthy, affordable, and sustainable.

## 11. Future Directions

Dairy proteins can be further advanced through process optimization and consumer education to better meet the consumer perception of sustainability. There are exceptional opportunities in the research and development of novel value-added dairy protein ingredients, like LF and BAPs. The category of alternative proteins is still emerging, and much research is still needed. First of all, regulation and labeling are crucial for every country, including current and relevant safety assessments and definitions and consensus on the use of terms like “meat” or “meat alternatives”, as well as regulations on labeling products “vegan” and “animal-free”. Secondly, sensory properties are a common challenge for alternative proteins and a major obstacle for consumer acceptance. Product development is needed to improve the flavor, texture, and appearance, while more research is needed to investigate the consumer perception of alternative-protein products. Moreover, the environmental impacts of alternative proteins need to be assessed to better understand their actual sustainability benefits. For precision fermentation proteins, cell-cultured proteins, algal proteins, and mycoproteins, more research on the scaling up of the manufacturing processes and improving the functionalities is needed to produce commercially profitable products that are accessible and affordable to consumers. Furthermore, many consumers lack knowledge on proteins and the related food technologies, so investigations on how to better educate and market both dairy and alternative-protein products to consumers are needed.

## Figures and Tables

**Table 1:** Inclusion and exclusion criteria used for article selection.

Inclusion Criteria	Exclusion Criteria
Protein ingredients (dairy/milk protein, plant proteins, precision fermentation protein, cell-cultured protein) are the primary part or a part of the study.	Publications that were not peer-reviewed
Studies must cover at least one of the following topics: definition, composition, processing, regulation, market, safety, protein quality nutrition, diseases risks, functional properties, applications, sensory properties, and consumer perception.	Studies published before 2000
Studies published in scientific journals	
Studies published in English	
Studies based on primary data	
Studies of subjects within the following categories: agriculture, food studies, environmental sciences, health sciences, public policy and administration, science and technology, and sustainability.	

**Table 2:** Published PDCAAS values of proteins.

Types	Source	PDCAAS	References
Dairy	Casein	1.00	Schaafsma, 2005 [90]
	Milk	1.00	Marinangeli and House, 2017 [91]
	Whey	1.00	Huang et al., 2018 [92]
Plants	Almond	0.39	Marinangeli and House, 2017 [93]
	Black beans	0.72	Schaafsma, 2005 [90]
	Chickpeas	0.74	Marinangeli and House, 2017 [91]
	Green lentils	0.63	Marinangeli and House, 2017 [91]
	Green peas	0.50	Nosworthy et al., 2017 [93]
	Navy beans	0.67	Marinangeli and House, 2017 [91]
	Oats	0.82	Marinangeli and House, 2017 [91]
	Red kidney beans	0.55	Nosworthy et al., 2017 [93]
	Red lentils	0.54	Marinangeli and House, 2017 [91]
	Soy protein concentrate	1.00	van den Berg et al., 2022 [94]
	Soy protein isolate	1.00	Hughes et al., 2011; Huang et al., 2018 [95,96]
	Soybeans	0.82	van den Berg et al., 2022 [94]
	Sunflower seeds	0.66	Marinangeli and House, 2017 [91]
	White rice	0.56	Nosworthy et al., 2017 [93]
	Yellow peas	0.64	Nosworthy et al., 2017 [93]
Algae	*Acutodesmus obliquus*	0.46	Wang et al., 2020 [97]
	*Alaria esculenta*	0.59	De Bhowmick and Hayes, 2022 [98]
	*Arthrospira platensis*	0.84	Palinska and Krumbein, 2000 [99]
	*Asparagopsis taxiformis*	0.31	De Bhowmick and Hayes, 2022 [98]
	*Chlorella sorokiniana*	0.81	Takeda, 1998; Wang et al., 2020 [97,100]
	*Chlorella vulgaris*	0.77	Rodrigues and da Silva Bon, 2011; Wang et al., 2020 [97,101]
	*Fucus serratus*	0.63	De Bhowmick and Hayes, 2022 [98]
	*Fucus vesiculosus*	0.08	De Bhowmick and Hayes, 2022 [98]
	*Hermetia illucens* L.	0.75	Traksele et al., 2021 [102]
	*Nannochloropsis oceanica*	0.36	Eilam et al., 2023 [103]
	*Palmaria palmata*	0.69	De Bhowmick and Hayes, 2022 [98]
	*Porphyra columbina*	0.33	Cian et al., 2014 [104]
	*Scenedesmus obliquus*	0.29	Williamson et al., 2023 [105]
	*Ulva lactuca*	0.15	De Bhowmick and Hayes, 2022 [98]
Fungi	*Fusarium venenatum*	1.00	Edwards and Cummings, 2010 [37]

**Table 3:** Flavor attributes of proteins.

Protein Source	Sample Evaluated ^1^	Flavor Attributes	References
Milk proteins	Rehydrated proteins	Cooked/milky, sweet aromatic, cereal, tortilla, brothy, cardboard, animal, sweet taste, astringent	Drake et al., 2003; Drake et al., 2014 [42,117]
Whey proteins	Rehydrated proteins	Sweet aromatic, cooked/milky, doughy/fatty/pasta, fatty, metallic, cucumber, brothy, cabbage, cardboard, animal, soapy, bitter, astringent	Drake et al., 2003; Karagul-yuceer et al., 2003; Carunchia et al., 2005; Wright et al., 2006 [113,117,118,119]
Caseins	Rehydrated proteins	Cooked/milky, sweet aromatic, potato/brothy, animal, cardboard, metallic, vitamin, sweet, bitter, astringent	Drake et al., 2003; Karagul-yuceer et al., 2003 [117,118]
Wheat	Whole wheat bread	Beany, grain, yeasty, bitter, sweet aromatic, sweet taste	Shogren et al., 2003 [120]
	Rehydrated proteins	Sweet aromatic, cereal/grain, cardboard, malty, sulfur, green/grassy, nutty, painty, cooked cereal/grain, bitter, sour, astringent	Chen et al., 1991 [121]
Corn	Corn meal extrudates	Raw flour, boiled corn, toasted corn, sweet aromatic, sweet taste, bitter	Chen et al., 1991 [121]
Barley	Barley pasta	Semolina, cooked, barley, sweet taste, bitter, astringent	Sinesio et al., 2008 [122]
Oats	Germinated, dried oats	Cereal, roasted, moist, musty, earthy, nutty, germ-like, rancid, sweet, bitter	Heinio et al., 2001 [123]
Rice	Enzymatic hydrolyzed rice bran protein concentrate	Rice bran, cereal, nut, milk powder, sweet aromatic, cocoa, feed, seafood, soy sauce	Arsa and Theerakulkait, 2018 [124]
	Rehydrated proteins	Sweet aromatic, cereal/grain, cardboard, fecal, nutty, painty, cooked cereal/grain, oxidized, bitter, sandy, astringent	Nishku, 2020 [125]
Quinoa	Ground native and malted quinoa	Butter, boiled vegetable, green, malty, musty	Almaguer et al., 2022 [126]
Buckwheat	Buckwheat-enriched pasta	Cereal, wheat, buckwheat, bitter	Škrobot et al., 2022 [127]
Chia	Rehydrated proteins	Sweet aromatic, green/grassy, painty, fruity, sandy, astringent	Nishku, 2020 [125]
Soybean	Rehydrated proteins	Sweet aromatic, cereal/grain, cardboard, doughy, nutty, beany, fruity, oxidized, salty, bitter, umami, astringent	Nishku, 2020 [125]
	Rehydrated protein isolates	Sweet aromatic, cereal, cardboard, brothy, roasted, malty, flour paste, sweet, fecal, bitter, astringent	Russell et al., 2006 [128]
Peas	Rehydrated proteins	Sweet aromatic, malty, pyrazine, sulfur, cereal/grain, cardboard, fecal, green pea, cheesy, doughy, nutty, beany/yellow pea, green/grassy, burnt, salty, bitter, umami, sandy, astringent	Nishku, 2020; Liu et al., 2023 [125,129]
Faba bean	Rehydrated proteins	Sweet aromatic, cereal/grain, pyrazine, sulfur, green/grassy, beany, metallic, fruity, bitter, astringent	Nishku, 2020 [125]
	Extrudates	Pea, oxidized, cereal, cooked pea, grass, yeast, veggie stock, umami, sweet, bitter	Tuccillo et al., 2022 [130]
Lupins	Protein isolates	Green/grassy, legume, solvent, cardboard, bitter, astringent	Bader et al., 2011 [131]
Mung bean	Rehydrated proteins	Green/grassy, woody, beany, seaweed, bitter, astringent	Nishku, 2020 [125]
Potato	Rehydrated proteins	Cereal/grain, cardboard, malty, green/grassy, doughy, seaweed, potato, bitter, sour, sandy, astringent	Nishku, 2020 [125]
Rapeseed	Protein isolates	Sweet aromatic, fruity, green, waxy, floral, woody, fatty, herbal, fresh, nutty	Chen et al., 2024 [132]
Peanut	Raw peanut, roasted peanut	Acidic, grain, nutty, burnt, fruity, grassy	Liu et al., 2022 [133]
Hemp seed	Rehydrated proteins	Sweet aromatic, cardboard, green pea, green/grassy, beany, earthy/soil, bitter, umami, sandy, astringent	Nishku, 2020 [125]
Sacha Inchi	Rehydrated proteins	Sweet aromatic, cereal/grain, cardboard, malty, green pea, pyrazine, green/grassy, nutty, beany, bitter, umami, sandy, astringent	Nishku, 2020 [125]
Pumpkin	Rehydrated proteins	Cardboard, fecal, woody, nutty, tortilla, umami, sandy, astringent	Nishku, 2020 [125]
Microalgae	Alga paste	grassy/vegetable/cucumber, cooked shrimp/seafood, fresh marine/fishy, rancid/fatty, fruity	Durme et al., 2013 [134]

^1^ A related application was presented if no information was found on the corresponding protein powder.

## Data Availability

No new data were created or analyzed in this study. Data sharing is not applicable to this article.
